# Telitacicept for systemic lupus erythematosus-associated peripheral neuropathy: a case report

**DOI:** 10.3389/fimmu.2025.1715983

**Published:** 2026-01-07

**Authors:** Jinhui Tan, Hai Huang, Linghua Tan, Bo Li, Ximei Wu, Ruonan She, Junjia Luo, Haitao Yang, Haoru Zhang

**Affiliations:** 1 People's Hospital of Longhua, Shenzhen, Guangdong, China; 2 Department of Health Management, People's Hospital of Longhua, Shenzhen, Guangdong, China; 3 Department of Health Management, Jiangmen Wuyi Hospital of Chinese Medicine, Jiangmen, Guangdong, China

**Keywords:** a proliferation-inducing ligand, B-cell lymphocyte stimulator, peripheral neuropathy, systemic lupus erythematosus, telitacicept

## Abstract

Peripheral neuropathy (PN) is a challenging manifestation of systemic lupus erythematosus (SLE) with limited evidence-based treatment guidelines. Current standard therapies, including glucocorticoids (GCs) and cyclophosphamide (CYC), are often effective but carry significant risks, such as gonadal toxicity with CYC, which is a major concern for young women. This case report describes the successful use of telitacicept, a novel dual inhibitor of B-lymphocyte stimulator (BLyS) and a proliferation-inducing ligand (APRIL), in a 37-year-old female with SLE-associated PN. The patient had a 17-year history of SLE and lupus nephritis, previously treated with high cumulative doses of CYC (13.2g), GCs, and mycophenolate mofetil (MMF). She developed PN in 2023, confirmed by electromyography showing axonal and demyelinating lesions. Due to her age and fertility concerns, subcutaneous telitacicept (160 mg/week) was added to her ongoing regimen of prednisone (10 mg/day) and MMF. Following telitacicept initiation, the patient’s neuropathic symptoms (numbness and hypoesthesia) completely resolved within months. Inflammatory markers (ESR, hs-CRP) and complement levels normalized, and proteinuria decreased. This clinical improvement allowed for a significant reduction in prednisone to 2.5 mg/day and MMF dosage. A follow-up electromyography eight months later showed no abnormalities. To our knowledge, this is the first report of telitacicept use for SLE-PN. It demonstrates that telitacicept can be a highly effective and steroid-sparing therapy, offering a safer alternative for patients where conventional immunosuppressants like CYC are contraindicated, particularly those of reproductive age. Future studies should explore parallels with immune checkpoint inhibitor (ICI)-related neuropathies.

## Introduction

Systemic lupus erythematosus (SLE) is a multisystem autoimmune disorder characterized by the production of diverse autoantibodies and immune complexes, which can lead to damage in multiple organ systems. Neuropsychiatric events in SLE are quite common, affecting up to 5% of the patients having other systemic manifestations (1). The reported frequency of peripheral nervous system (PNS) involvement in SLE patients with neuropsychiatric (NP) manifestations ranges from 4.9% to 7.6% (2, 3). A 2019 cohort study involving 1827 patients with NPSLE found that PNS manifestations occurred in 7.6% of participants, with polyneuropathies (PNP) being the most common (41%), followed by single and multiplex mononeuropathies (MNP) (27.3%) (2). Electrophysiological studies revealed axonal damage in 41.7% and demyelination in 21.7% of patients diagnosed with PNP or MNP (2). It is important to note that no universally accepted consensus or clear guidelines currently exist regarding the treatment of SLE-associated peripheral neuropathy (PN). According to the European Alliance of Associations for Rheumatology (EULAR), glucocorticoids (GCs)—either alone or in combination with other immunosuppressive agents—are recommended for managing SLE-associated PN, while interventions such as plasmapheresis, intravenous immunoglobulins, and rituximab are generally reserved for severe cases (1). These EULAR recommendations are primarily derived from studies focusing broadly on neuropsychiatric manifestations of SLE rather than specifically on PNS involvement. Although one randomized clinical trial (RCT) was referenced, it included only seven patients with PN (4). Despite the limited direct evidence, the strength of the recommendation was graded as A, supported by a category 1 level of evidence (4–9).

B cells play a central role in the pathogenesis of SLE (10), as their dysregulated activation, differentiation, and survival result in the secretion of autoantibodies that contribute to systemic tissue injury. The cytokines B-lymphocyte stimulator (BLyS) and a proliferation-inducing ligand (APRIL) are critically involved in regulating B-cell maturation and differentiation processes (11). Telitacicept is a significant biologic agent that potently inhibits the proliferation, differentiation, and survival of B cells and plasma cells through specific dual targeting of BLyS and APRIL. It also demonstrates potential in suppressing the activity of long-lived plasma cells (12). Although approved in China in March 2021 for treating SLE (13), its broader clinical adoption has been constrained by high treatment costs.

This case report details a 37-year-old female patient with SLE, initially diagnosed in 2008, who developed PN in 2023—15 years after the SLE diagnosis. The patient achieved complete remission following telitacicept therapy, providing the first evidence of its efficacy in SLE-PN. Recent studies also support telitacicept’s role in neurological complications post-immune checkpoint inhibitors (ICIs), such as neuropathy, suggesting a broader application in immune-mediated neuropathies (14, 15). To the best of our knowledge, this is the first reported case of a patient with SLE-associated PN who received telitacicept treatment.

## Case report

**Figure 1:**
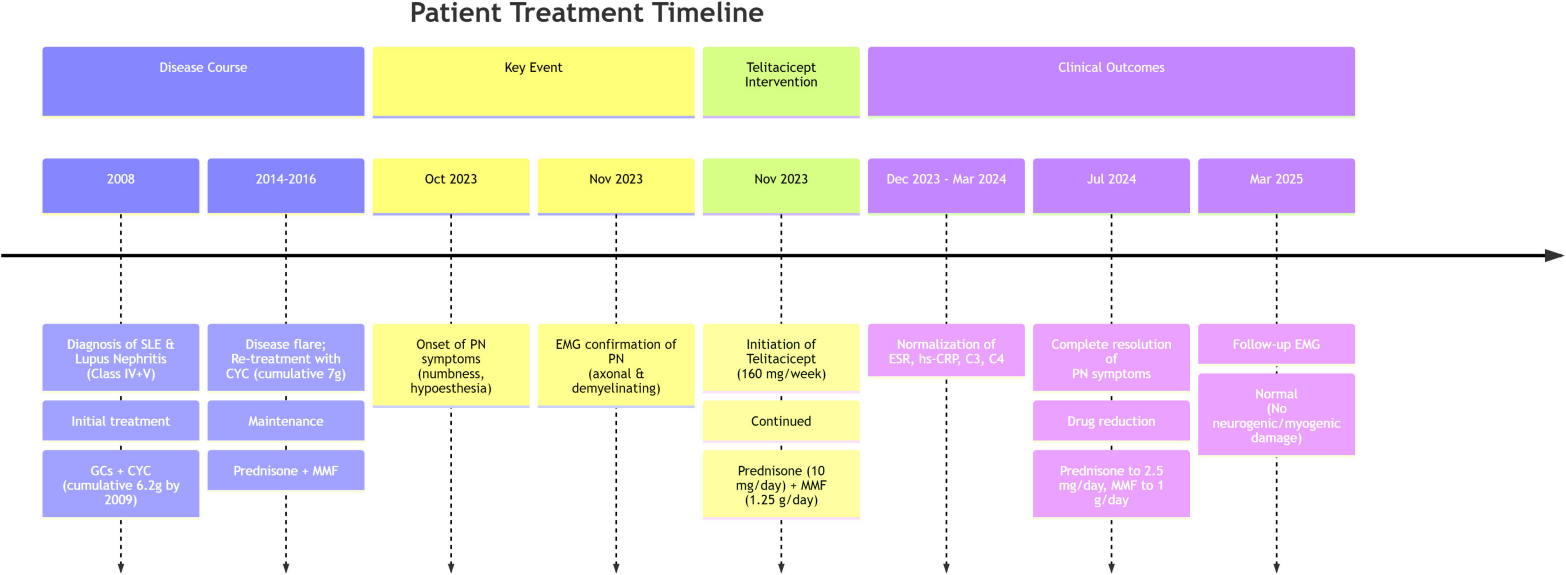
Treatment timeline and clinical course of a patient with SLE-associated peripheral neuropathy treated with telitacicept.PN, peripheral neuropathy; SLE, systemic lupus erythematosus; LN, lupus nephritis; GCs, glucocorticoids; CYC, cyclophosphamide; MMF, mycophenolate mofetil; EMG, electromyography; ESR, erythrocyte sedimentation rate; hs-CRP, high-sensitivity C-reactive protein.

The patient is a 37-year-old unmarried woman without children. **Figure 1** illustrates the clinical timeline, summarizing key events from 2008 to 2025, including disease course, interventions, and outcomes. Seventeen years ago (on June 27, 2008), she presented with facial erythema and edema without any obvious precipitating factors, accompanied by pain in both hands and knees. She sought medical attention at Wuhan Union Hospital (Wuhan, China), where examinations revealed decreased complement levels, positive ANA, anti-dsDNA antibody, and anti-Sm antibody results, along with 3+ proteinuria. A renal biopsy was performed, and the pathology indicated lupus nephritis (LN) (types IV and V). The diagnosis was SLE with LN. She was treated with GCs. On August 24, 2008, she commenced monthly cyclophosphamide (CYC) injections at a dose of 1 gram per injection, accumulating a total of 6.2 grams over one year. Subsequently, her urine protein test turned negative, and she discontinued CYC injections. During this period, she only took oral prednisone, with the dosage gradually tapered to 10 milligrams once daily. Eleven years ago (on March 20, 2014), due to the recurrence of positive urine protein, she visited Futian Xiangmihu Rheumatism Hospital (Shenzhen, China) and resumed CYC injections every two weeks at a dose of 0.4-0.6 grams per injection. After her urine protein test turned negative again, she discontinued CYC. The last injection was administered on January 7, 2016, with a cumulative dose of 7 grams.

Seven years ago, the patient visited Peking University Shenzhen Hospital (Shenzhen, China) due to aggravated joint pain. The anti-dsDNA antibody test returned positive at 319.8 IU/ml, and the ANA test showed a 1:320 titer with granular and nucleolar patterns. The urine protein test was suspected to be positive. The dosage of prednisone was increased to 15 mg once daily, and mycophenolate mofetil was prescribed at 0.75 g in the morning and 0.5 g in the evening. The patient’s joint pain subsequently improved, allowing for a reduction in prednisone to 10 mg once daily.

**Figure 2:**
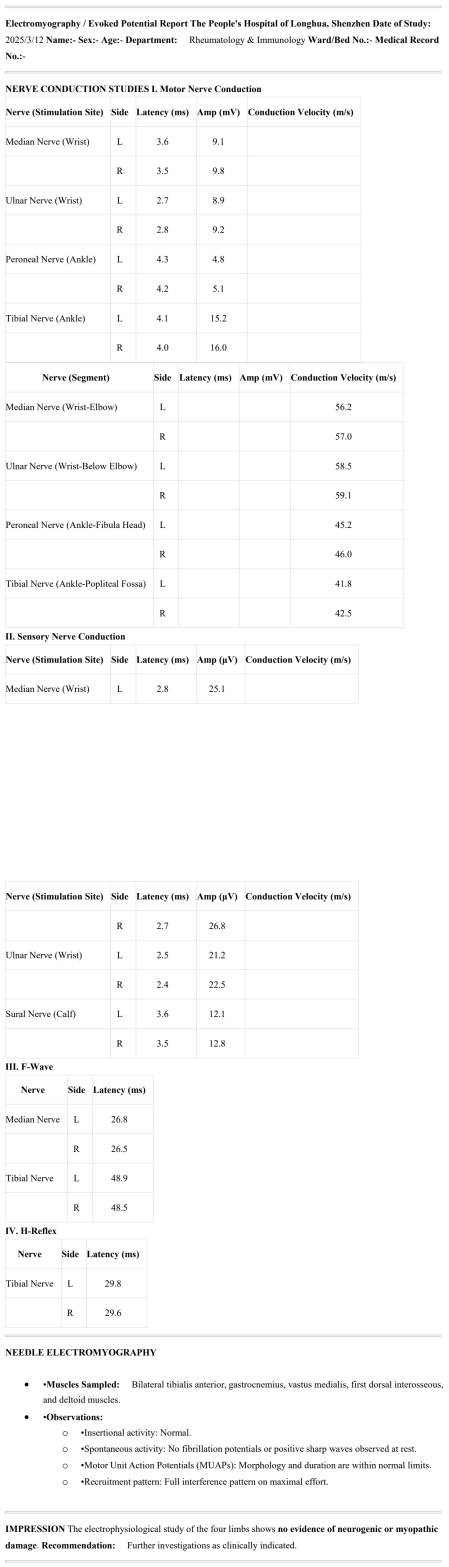
Electromyography report showing nerve conduction study results and needle electromyography findings.

In October 2023, the patient presented with numbness and hypoesthesia localized to the right knee and lower leg, without motor weakness, muscle atrophy, or reflex abnormalities. No other focal neurological signs were observed. From November 2 to 8, 2023, the patient was hospitalized at Guangzhou University of Chinese Medicine Shenzhen Hospital (Shenzhen, China). Blood tests revealed an erythrocyte sedimentation rate (ESR) of 54 mm/h, a high-sensitivity C-reactive protein (hs-CRP) level of 14.25 mg/L, Complement 3 (C3) at 0.69 g/L, C4 at 0.09 g/L (**Table 1**), and a positive c-ANCA result. However, a confirmatory PR3-ANCA ELISA test was negative, and systemic evaluation (e.g., renal function, urinalysis, chest X-ray) showed no evidence of vasculitis, consistent with SLE without a separate vasculitic process. The 24-hour urine protein excretion was measured at 179.8 mg. Nerve-electromyography in the limbs indicated: 1. Segmental lesions in the bilateral ulnar nerves at the elbow, characterized by motor fiber involvement and demyelinating changes; 2. Peripheral lesions in the right superficial peroneal nerve, with sensory fiber involvement and axonal damage, along with myogenic damage in the right anterior tibial muscle and right vastus medialis muscle (data not shown). It is essential to provide patients with a comprehensive and detailed explanation of the advantages and disadvantages of the treatment plan. For instance, continuous use of CYC pulse therapy may lead to premature ovarian failure due to the excessive accumulation of CYC in the body (16–19). High-dose glucocorticoid therapy may cause adverse reactions such as moon face, buffalo hump, osteoporosis, and gastrointestinal ulcers (20–22). When treated with Benlysta, patients are required to be hospitalized regularly for infusion therapy (23). In contrast, telitacicept avoids the side effects associated with CYC and high-dose GCs previously mentioned (13). Patients can simply receive treatment in outpatient settings, but it requires weekly administration and comes at a relatively higher cost. The patient continued oral administration of mycophenolate mofetil at 0.75 g in the morning and 0.5 g in the evening, with prednisone maintained at 10 mg once daily. On November 8, 2023, subcutaneous injections of telitacicept were initiated at a dose of 160 mg once weekly (13). The patient did not receive concomitant therapies such as IVIG, vitamin supplements, gabapentin, or other immunomodulators during telitacicept treatment. The patient began receiving medical treatment at People's Hospital of Longhua, Shenzhen (Shenzhen, China) on November 15, 2023, and continued the previous treatment plan. The numbness and hypoesthesia in the right knee and right lower leg gradually improved, and subsequent blood tests showed that the ESR, hs-CRP, C3, and C4 levels normalized (**Table 1**). By July 18, 2024, the patient experienced complete resolution of the numbness and hypoest in the right knee and right lower leg. Although no formal patient-reported outcome scale was administered, her subjective symptom diary and clinical evaluations consistently indicated progressive recovery. The administration of telitacicept was then adjusted to every three weeks at a dose of 160 mg, prednisone was reduced to 2.5 mg once daily, and MMF was decreased to 0.5 g twice daily. On March 12, 2025, a follow-up nerve-electromyography in the limbs showed no evidence of neurogenic or myogenic damage (**Figure 2**). Due to the absence of assessment for peripheral neuropathy in the SLEDAI-2K scoring system, the patient experienced peripheral neuropathy in 2023, which was accompanied by hypocomplementemia (score 2), but did not show symptoms like arthritis, proteinuria, rash, alopecia, or oral ulcers. Hence, the score for this stage was set at 2. Following treatment, the aforementioned symptoms improved, resulting in a score reduction to 0.

**Table 1:** Changes in ESR, hs-CRP, C3, and C4 levels before and after treatment with telitacicept.

Index	2023-11-02	2023-12-13	2024-03-13	2025-02-13	2025-03-11
ESR (mm/h)	54	52	25	20	14
hs-CRP (mg/L)	14.25	0.7	0.6	0.73	0.8
C3 (g/L)	0.69	–	–	0.81	0.7
C4 (g/L)	0.09	–	–	0.13	0.1

The reference ranges for ESR, hs-CRP, C3, and C4 are 0–15 mm/h, 0–10 mg/L, 0.7-1.4 g/L, and 0.1-0.4 g/L, respectively. ESR, erythrocyte sedimentation rate; hs-CRP, high-sensitivity C-reactive protein; C3, complement 3; C4, complement 4; -, insufficient information.

## Discussion

SLE is a chronic autoimmune disorder characterized by multisystem involvement, including the PNS. PN, a common neurological manifestation of SLE, affects approximately 5-18% of patients and can present as mononeuropathy, polyneuropathy, or autonomic dysfunction, often leading to significant morbidity (3, 24, 25). The pathogenesis involves immune-mediated mechanisms such as vasculitis, autoantibody deposition (e.g., anti-dsDNA, anti-ribosomal P), and cytokine-driven inflammation, which damage nerve fibers (18, 26, 27). Treatment typically relies on immunosuppressive agents like GCs and CYC, but these are associated with substantial toxicities, particularly in young women of reproductive age (17, 20, 22). This case illustrates the challenges in managing SLE with peripheral neuropathy and highlights the emerging role of telitacicept, a novel BLyS/APRIL dual inhibitor, as an effective and safer alternative. We discuss the implications of GCs and CYC therapies, the rationale for telitacicept use, and its application in this patient, supported by evidence from recent literature.

GCs remain the first-line therapy for inducing remission in active SLE, including neuropsychiatric manifestations (28–30). They exert rapid anti-inflammatory and immunosuppressive effects by modulating gene expression, inhibiting cytokine production, and reducing immune cell activation (20–22). In this case, the patient received prednisone at doses up to 15 mg/day, which initially controlled disease activity but posed risks of long-term complications. High-dose or prolonged GCs use is linked to numerous adverse effects, including osteoporosis, avascular necrosis, hypertension, diabetes mellitus, Cushingoid features (e.g., moon face, buffalo hump), gastrointestinal ulcers, and increased infection susceptibility (31–33). For instance, GCs can cause hypothalamic-pituitary-adrenal axis suppression (34) and metabolic syndrome (35), impacting quality of life (21). The EULAR guidelines recommend minimizing GCs exposure by using the lowest effective dose (preferably ≤5 mg/day prednisone equivalent) and tapering rapidly once remission is achieved to reduce damage accrual (36, 37). However, many patients, like this one, experience flares requiring sustained GCs therapy, leading to irreversible organ damage (38–40). Steroid-sparing agents are thus crucial, and telitacicept offers a promising option by allowing GCs reduction, as seen in this case where prednisone was tapered to 2.5 mg/day after telitacicept initiation (27, 41, 42).

CYC is an alkylating agent used for severe SLE manifestations, such as LN and neuropsychiatric involvement (18), due to its potent immunosuppressive effects. However, its use is limited by dose-dependent toxicities, including myelosuppression, hemorrhagic cystitis, malignancies, and gonadal failure (19). Gonadal toxicity is particularly relevant for young women, as CYC can cause premature ovarian failure (POF), leading to infertility (16, 17, 19). Studies show that the risk of POF increases with cumulative CYC dose (>10 g) and older age at treatment initiation (17, 19). In this patient, a 37-year-old unmarried woman without children, the cumulative CYC dose reached 13.2 g over two treatment courses (6.2 g in 2008–2009 and 7 g in 2014-2016), significantly elevating her risk of POF. This risk necessitated a switch to a safer agent, as fertility preservation is a priority. Guidelines recommend gonadotropin-releasing hormone agonists (e.g., leuprolide) for ovarian protection during CYC therapy (16, 17), but these were not utilized here, underscoring the importance of individualized treatment plans. The transition to telitacicept avoided further gonadal damage, aligning with recommendations to minimize CYC exposure in women desiring fertility (12, 43).

Telitacicept is a recombinant fusion protein comprising the transmembrane activator and calcium modulator and cyclophilin ligand interactor (TACI) receptor fused to the Fc domain of human IgG1 (12, 13, 44). It inhibits both BLyS and APRIL, key cytokines involved in B-cell activation, differentiation, and autoantibody production (12, 13, 45). By neutralizing these cytokines, telitacicept reduces the survival of autoreactive B cells and plasma cells, modulating the autoimmune response in SLE (12, 45). In a phase 2b trial (NCT02885610), telitacicept demonstrated significant efficacy in reducing SLE disease activity, manifested by SLE Responder Index-4 (SRI-4) response rates of 71.0%, 68.3%, and 75.8% at week 48 for the 80 mg, 160 mg, and 240 mg weekly doses, respectively, compared to 33.9% with placebo (all p < 0.001) (41). It also allowed for GCs tapering and improved serological markers (e.g., reduction in anti-dsDNA antibodies and normalization of complement levels) (27, 41, 46). In this case, telitacicept 160 mg weekly led to the resolution of PN symptoms (numbness and hypoesthesia), normalized the inflammatory markers (ESR and hs-CRP), improved complement levels (C3 and C4), and reduced proteinuria, consistent with clinical trial outcomes (42, 47–49). The patient’s ability to taper prednisone to 2.5 mg/day and reduce MMF dosage highlights its steroid-sparing benefit (27, 42, 46).

Telitacicept offers several advantages over GCs and CYC (1): ​​Safety Profile​​: It has a favorable safety profile with low rates of serious infections and no gonadal toxicity, making it suitable for long-term use (49–51). In clinical trials, adverse events were mostly mild, such as upper respiratory infections, and no increased risk of malignancies was reported (49) (2). ​​Administration​​: Unlike CYC, which requires intravenous pulses and hospitalization, telitacicept is administered subcutaneously in outpatient settings, improving convenience and quality of life (49, 50) (3). ​​Fertility Preservation​​: By avoiding CYC-induced gonadal damage, telitacicept is ideal for young women desiring fertility, as in this case (17, 52) (4). ​​Efficacy in Neuropathy​​: Although data specifically on SLE-associated neuropathy are limited (49, 51), telitacicept’s broader B-cell targeting may address neuroinflammation driven by autoantibodies, as evidenced by improved nerve function in this patient. Real-world studies support its use in refractory SLE cases (49, 51).

PN in SLE results from vasculitic ischemia, axonal degeneration, or demyelination due to immune complex deposition and cytokine release (25, 53, 54). In this patient, electromyography revealed segmental ulnar nerve lesions and superficial peroneal nerve involvement, consistent with SLE-related neuropathy. Traditional treatments include high-dose GCs and CYC, but responses are variable, and relapses are common (18, 55). Telitacicept’s targeting of BLyS/APRIL may mitigate neuroinflammation by reducing autoantibody production and B-cell infiltration (49, 50, 56). The complete EMG normalization in this case, though uncommon in SLE-PN, may be attributed to early and effective B-cell targeting by telitacicept, which suppressed inflammation and facilitated neural repair. Future studies should focus on telitacicept’s role in neurological manifestations of SLE.

This case demonstrates the successful use of telitacicept in a young woman with SLE and PN, where concerns about CYC-induced infertility guided therapeutic choice. Telitacicept provided effective disease control, GCs sparing, and a favorable safety profile. Future research should explore telitacicept’s long-term effects on fertility, quality of life, and neuropathy outcomes. As personalized medicine advances, telitacicept may become a first-line option for SLE patients prioritizing fertility and minimizing steroid exposure.

## Data Availability

The original contributions presented in the study are included in the article/supplementary material. Further inquiries can be directed to the corresponding author.

## References

[1] Bertsias GK Ioannidis JP Aringer M Bollen E Bombardieri S Bruce IN . EULAR recommendations for the management of systemic lupus erythematosus with neuropsychiatric manifestations: report of a task force of the EULAR standing committee for clinical affairs. Ann Rheum Dis. (2010) 69:2074–82. doi: 10.1136/ard.2010.130476, PMID: 20724309

[2] Hanly JG Li Q Su L Urowitz MB Gordon C Bae SC . Peripheral nervous system disease in systemic lupus erythematosus: results from an international inception cohort study. Arthritis Rheumatol. (2020) 72:67–77. doi: 10.1002/art.41070, PMID: 31390162 PMC6935421

[3] Bortoluzzi A Piga M Silvagni E Chessa E Mathieu A Govoni M . Peripheral nervous system involvement in systemic lupus erythematosus: a retrospective study on prevalence, associated factors and outcome. Lupus. (2019) 28:465–74. doi: 10.1177/0961203319828499, PMID: 30739544

[4] Barile-Fabris L Ariza-Andraca R Olguín-Ortega L Jara LJ Fraga-Mouret A Miranda-Limón JM . Controlled clinical trial of IV cyclophosphamide versus IV methylprednisolone in severe neurological manifestations in systemic lupus erythematosus. Ann Rheum Dis. (2005) 64:620–5. doi: 10.1136/ard.2004.025528, PMID: 15769918 PMC1755456

[5] Stojanovich L Stojanovich R Kostich V Dzjolich E . Neuropsychiatric lupus favourable response to low dose i.v. cyclophosphamide and prednisolone (pilot study). Lupus. (2003) 12:3–7. doi: 10.1191/0961203303lu251oa, PMID: 12587819

[6] Neuwelt CM . The role of plasmapheresis in the treatment of severe central nervous system neuropsychiatric systemic lupus erythematosus. Ther Apher Dial. (2003) 7:173–82. doi: 10.1046/j.1526-0968.2003.00032.x, PMID: 12918940

[7] Bartolucci P Bréchignac S Cohen P Le Guern V Guillevin L . Adjunctive plasma exchanges to treat neuropsychiatric lupus: a retrospective study on 10 patients. Lupus. (2007) 16:817–22. doi: 10.1177/0961203307081840, PMID: 17895305

[8] Levy Y Sherer Y Ahmed A Langevitz P George J Fabbrizzi F . A study of 20 SLE patients with intravenous immunoglobulin–clinical and serologic response. Lupus. (1999) 8:705–12. doi: 10.1191/096120399678841007, PMID: 10602441

[9] Milstone AM Meyers K Elia J . Treatment of acute neuropsychiatric lupus with intravenous immunoglobulin (IVIG): a case report and review of the literature. Clin Rheumatol. (2005) 24:394–7. doi: 10.1007/s10067-004-1046-9, PMID: 15662488

[10] Ma K Du W Wang X Yuan S Cai X Liu D . Multiple functions of B cells in the pathogenesis of systemic lupus erythematosus. Int J Mol Sci. (2019) 20:6021. doi: 10.3390/ijms20236021, PMID: 31795353 PMC6929160

[11] Möckel T Basta F Weinmann-Menke J Schwarting A . B cell activating factor (BAFF): Structure, functions, autoimmunity and clinical implications in Systemic Lupus Erythematosus (SLE). Autoimmun Rev. (2021) 20:102736. doi: 10.1016/j.autrev.2020.102736, PMID: 33333233

[12] Shi F Xue R Zhou X Shen P Wang S Yang Y . Telitacicept as a BLyS/APRIL dual inhibitor for autoimmune disease. Immunopharmacol Immunotoxicol. (2021) 43:666–73. doi: 10.1080/08923973.2021.1973493, PMID: 34519594

[13] Dhillon S . Telitacicept: first approval. Drugs. (2021) 81:1671–5. doi: 10.1007/s40265-021-01591-1, PMID: 34463932

[14] Rossi S Farina A Malvaso A Dinoto A Fionda L Cornacchini S . Clinical course of neurologic adverse events associated with immune checkpoint inhibitors: focus on chronic toxicities. Neurol Neuroimmunol Neuroinflamm. (2024) 11:e200314. doi: 10.1212/NXI.0000000000200314, PMID: 39298719 PMC11413993

[15] Malvaso A Giglio P Diamanti L Gastaldi M Vegezzi E Pace A . Unravelling the acute, chronic and steroid-refractory management of high-grade neurological immune-related adverse events: A call to action. Brain Sci. (2024) 14:764. doi: 10.3390/brainsci14080764, PMID: 39199458 PMC11352216

[16] Raptopoulou A Sidiropoulos P Boumpas D . Ovarian failure and strategies for fertility preservation in patients with systemic lupus erythematosus. Lupus. (2004) 13:887–90. doi: 10.1002/14651858.CD002265.pub3, PMID: 15645741

[17] Giambalvo S Garaffoni C Silvagni E Furini F Rizzo R Govoni M . Factors associated with fertility abnormalities in women with systemic lupus erythematosus: a systematic review and meta-analysis. Autoimmun Rev. (2022) 21:103038. doi: 10.1016/j.autrev.2022.103038, PMID: 34995765

[18] Fernandes Moça Trevisani V Castro AA Ferreira Neves Neto J Atallah AN . Cyclophosphamide versus methylprednisolone for treating neuropsychiatric involvement in systemic lupus erythematosus. Cochrane Database Syst Rev. (2013) 2013:Cd002265. doi: 10.1002/14651858.CD002265, PMID: 23450535 PMC6823222

[19] Petri M . Cyclophosphamide: new approaches for systemic lupus erythematosus. Lupus. (2004) 13:366–71. doi: 10.1191/0961203303lu1028oa, PMID: 15230294

[20] Huang S Jia Y Zhang Y Chen H Deng C Fei Y . Effects of glucocorticoid withdrawal on relapse risk in systemic lupus erythematosus: A systematic review and meta-analysis. iScience. (2025) 28:112875. doi: 10.1016/j.isci.2025.112875, PMID: 40662196 PMC12256336

[21] Martin-Iglesias D Paredes-Ruiz D Ruiz-Irastorza G . Use of glucocorticoids in SLE: A clinical approach. Mediterr J Rheumatol. (2024) 35:342–53. doi: 10.31138/mjr.230124.uos, PMID: 39193186 PMC11345604

[22] Mok TC Mok CC . Glucocorticoid in systemic lupus erythematosus: the art beyond science. Expert Rev Clin Immunol. (2025) 21:543–53. doi: 10.1080/1744666X.2025.2494654, PMID: 40232132

[23] Blair HA Duggan ST . Belimumab: A review in systemic lupus erythematosus. Drugs. (2018) 78:355–66. doi: 10.1007/s40265-018-0872-z, PMID: 29396833

[24] Omdal R Henriksen OA Mellgren SI Husby G . Peripheral neuropathy in systemic lupus erythematosus. Neurology. (1991) 41:808–11. doi: 10.1212/WNL.41.6.808, PMID: 1646422

[25] Oomatia A Fang H Petri M Birnbaum J . Peripheral neuropathies in systemic lupus erythematosus: clinical features, disease associations, and immunologic characteristics evaluated over a twenty-five-year study period. Arthritis Rheumatol. (2014) 66:1000–9. doi: 10.1002/art.38302, PMID: 24757151

[26] Bougea A Anagnostou E Konstantinos G George P Triantafyllou N Kararizou E . A systematic review of peripheral and central nervous system involvement of rheumatoid arthritis, systemic lupus erythematosus, primary sjögren’s syndrome, and associated immunological profiles. Int J Chronic Dis. (2015) 910352:2015. doi: 10.1155/2015/910352, PMID: 26688829 PMC4673346

[27] Jin HZ Li YJ Wang X Li Z Ma B Niu L . Efficacy and safety of telitacicept in patients with systemic lupus erythematosus: a multicentre, retrospective, real-world study. Lupus Sci Med. (2023) 10:e001074. doi: 10.1136/lupus-2023-001074, PMID: 38007228 PMC10679987

[28] Fanouriakis A Kostopoulou M Alunno A Aringer M Bajema I Boletis JN . update of the EULAR recommendations for the management of systemic lupus erythematosus. Ann Rheum Dis. (2019) 78:736–45:2019. doi: 10.1136/annrheumdis-2019-215089, PMID: 30926722

[29] Bertsias GK Tektonidou M Amoura Z Aringer M Bajema I Berden JH . Joint European League Against Rheumatism and European Renal Association-European Dialysis and Transplant Association (EULAR/ERA-EDTA) recommendations for the management of adult and paediatric lupus nephritis. Ann Rheum Dis. (2012) 71:1771–82. doi: 10.1136/annrheumdis-2012-201940, PMID: 22851469 PMC3465859

[30] Hahn BH McMahon MA Wilkinson A Wallace WD Daikh DI Fitzgerald JD . American College of Rheumatology guidelines for screening, treatment, and management of lupus nephritis. Arthritis Care Res (Hoboken). (2012) 64:797–808. doi: 10.1002/acr.21664, PMID: 22556106 PMC3437757

[31] Alarcón GS Ugarte-Gil MF Pons-Estel G Vilá LM Reveille JD McGwin G Jr . Remission and low disease activity state (LDAS) are protective of intermediate and long-term outcomes in SLE patients. Results from LUMINA (LXXVIII), a multiethnic, multicenter US cohort. Lupus. (2019) 28:423–6. doi: 10.1177/0961203319826693, PMID: 30678605

[32] Zen M Iaccarino L Gatto M Bettio S Nalotto L Ghirardello A . Prolonged remission in Caucasian patients with SLE: prevalence and outcomes. Ann Rheum Dis. (2015) 74:2117–22. doi: 10.1136/annrheumdis-2015-207347, PMID: 26223434

[33] Lateef A Petri M . Management of pregnancy in systemic lupus erythematosus. Nat Rev Rheumatol. (2012) 8:710–8. doi: 10.1038/nrrheum.2012.133, PMID: 22907290

[34] Andrews MH Wood SA Windle RJ Lightman SL Ingram CD . Acute glucocorticoid administration rapidly suppresses basal and stress-induced hypothalamo-pituitary-adrenal axis activity. Endocrinology. (2012) 153:200–11. doi: 10.1210/en.2011-1434, PMID: 22087024 PMC3279736

[35] Dunford EC Riddell MC . The metabolic implications of glucocorticoids in a high-fat diet setting and the counter-effects of exercise. Metabolites. (2016) 6:44. doi: 10.3390/metabo6040044, PMID: 27929385 PMC5192450

[36] Duru N van der Goes MC Jacobs JW Andrews T Boers M Buttgereit F . EULAR evidence-based and consensus-based recommendations on the management of medium to high-dose glucocorticoid therapy in rheumatic diseases. Ann Rheum Dis. (2013) 72:1905–13. doi: 10.1136/annrheumdis-2013-203249, PMID: 23873876

[37] Bergstra SA Sepriano A Kerschbaumer A van der Heijde D Caporali R Edwards CJ . Efficacy, duration of use and safety of glucocorticoids: a systematic literature review informing the 2022 update of the EULAR recommendations for the management of rheumatoid arthritis. Ann Rheum Dis. (2023) 82:81–94. doi: 10.1136/ard-2022-223358, PMID: 36410794

[38] Tang XL Zhu TY Hung VW Qin L Wong CK Kun EW . Increased organ damage associated with deterioration in volumetric bone density and bone microarchitecture in patients with systemic lupus erythematosus on longterm glucocorticoid therapy. J Rheumatol. (2012) 39:1955–63. doi: 10.3899/jrheum.120213, PMID: 22896029

[39] Sprangers B Monahan M Appel GB . Diagnosis and treatment of lupus nephritis flares–an update. Nat Rev Nephrol. (2012) 8:709–17. doi: 10.1038/nrneph.2012.220, PMID: 23147758

[40] Gatto M Frontini G Calatroni M Reggiani F Depascale R Cruciani C . Effect of sustained clinical remission on the risk of lupus flares and impaired kidney function in patients with lupus nephritis. Kidney Int Rep. (2024) 9:1047–56. doi: 10.1016/j.ekir.2024.01.016, PMID: 38765576 PMC11101726

[41] Wu D Li J Xu D Merrill JT van Vollenhoven RF Liu Y . Telitacicept in patients with active systemic lupus erythematosus: results of a phase 2b, randomised, double-blind, placebo-controlled trial. Ann Rheum Dis. (2024) 83:475–87. doi: 10.1136/ard-2023-224854, PMID: 38129117 PMC10958275

[42] Ji L Geng Y Zhang X Deng X Song Z Tan M . B cell pathway dual inhibition for systemic lupus erythematosus: a prospective single-arm cohort study of telitacicept. MedComm (2020). (2024) 5:e515. doi: 10.1002/mco2.515, PMID: 38525109 PMC10960726

[43] Cheng J Peng Y Wu Q Wu Q He J Yuan G . Efficacy and safety of telitacicept therapy in systemic lupus erythematosus with hematological involvement. Clin Rheumatol. (2024) 43:2229–36. doi: 10.1007/s10067-024-06992-7, PMID: 38767710

[44] Fan Y Gao D Zhang Z . Telitacicept, a novel humanized, recombinant TACI-Fc fusion protein, for the treatment of systemic lupus erythematosus. Drugs Today (Barc). (2022) 58:23–32. doi: 10.1358/dot.2022.58.1.3352743, PMID: 35107091

[45] Zhao Q Chen X Hou Y Jiang J Zhong W Yao X . Pharmacokinetics, pharmacodynamics, safety, and clinical activity of multiple doses of RCT-18 in chinese patients with systemic lupus erythematosus. J Clin Pharmacol. (2016) 56:948–59. doi: 10.1002/jcph.686, PMID: 26634642

[46] Chen R Fu R Lin Z Huang C Huang W . The efficacy and safety of telitacicept for the treatment of systemic lupus erythematosus: a real life observational study. Lupus. (2023) 32:94–100. doi: 10.1177/09612033221141253, PMID: 36416639

[47] Chen Y Shi N Lei X Ren P Lan L Chen L . The efficacy of rituximab plus belimumab or telitacicept in refractory lupus nephritis. Rheumatol (Oxford). (2025) 64:221–7. doi: 10.1093/rheumatology/kead674, PMID: 38145455

[48] Ma X Fu X Cui B Lin H . Telitacicept for recalcitrant cutaneous manifestations of systemic lupus erythematosus: A case report and review of the literature. Tohoku J Exp Med. (2022) 258:219–23. doi: 10.1620/tjem.2022.J074, PMID: 36047131

[49] Fan Q Yang H Liu Y . Safety and efficacy of telitacicept in refractory systemic lupus erythematosus patients who failed treatment with belimumab: A case series. Z Rheumatol. (2024) 83:387–92. doi: 10.1007/s00393-023-01461-z, PMID: 38157053 PMC11147914

[50] Fang Z Zhang Y Zhang Y Zhang Q Qu X Pan S . Telitacicept as an alternative to non-steroidal immunosuppressive therapies in the treatment of myasthenia gravis: a study on clinical efficacy and steroid-sparing effect. Front Immunol. (2025) 16:1549034. doi: 10.3389/fimmu.2025.1549034, PMID: 40196119 PMC11973063

[51] Ren Y Chen S Yang H . Case Report: Telitacicept in treating a patient with NF155+ autoimmune nodopathy: a successful attempt to manage recurrent elevated sero-anti-NF155 antibodies. Front Immunol. (2023) 14:1279808. doi: 10.3389/fimmu.2023.1279808, PMID: 37965304 PMC10642300

[52] Petri M Bruce IN Dörner T Tanaka Y Morand EF Kalunian KC . Baricitinib for systemic lupus erythematosus: a double-blind, randomised, placebo-controlled, phase 3 trial (SLE-BRAVE-II). Lancet. (2023) 401:1011–9. doi: 10.1016/S0140-6736(22)02546-6, PMID: 36848919

[53] Toledano P Orueta R Rodríguez-Pintó I Valls-Solé J Cervera R Espinosa G . Peripheral nervous system involvement in systemic lupus erythematosus: Prevalence, clinical and immunological characteristics, treatment and outcome of a large cohort from a single centre. Autoimmun Rev. (2017) 16:750–5. doi: 10.1016/j.autrev.2017.05.011, PMID: 28483540

[54] Gwathmey KG Satkowiak K . Peripheral nervous system manifestations of rheumatological diseases. J Neurol Sci. (2021) 424:117421. doi: 10.1016/j.jns.2021.117421, PMID: 33824004

[55] Constantin A Năstase D Tulbă D Bălănescu P Băicuş C . Immunosuppressive therapy of systemic lupus erythematosus associated peripheral neuropathy: A systematic review. Lupus. (2020) 29:1509–19. doi: 10.1177/0961203320948181, PMID: 32757735

[56] Yao X Ren Y Zhao Q Chen X Jiang J Liu D . Pharmacokinetics analysis based on target-mediated drug distribution for RC18, a novel BLyS/APRIL fusion protein to treat systemic lupus erythematosus and rheumatoid arthritis. Eur J Pharm Sci. (2021) 159:105704. doi: 10.1016/j.ejps.2021.105704, PMID: 33440243

